# Special Issue: “Microbial Enzymes for Biotechnological Applications”

**DOI:** 10.3390/ijms26052040

**Published:** 2025-02-26

**Authors:** Salvatore Fusco, Martina Aulitto

**Affiliations:** 1 Biochemistry and Industrial Biotechnology (BIB) Laboratory, Department of Biotechnology, University of Verona, 37134 Verona, Italy; 2 Department of Biology, University of Naples Federico II, 80126 Naples, Italy; martina.aulitto@unina.it

Microbial biocatalysts are at the forefront of modern biotechnology, offering sustainable solutions to some of the world’s most pressing environmental and industrial challenges [1]. These powerful tools represent about 90% of the enzyme market, driven by their rapid growth, ease of manipulation, and versatile application potential compared to biocatalysts from plant and animal sources [2]. As the global push towards a circular economy accelerates, microbial enzymes are emerging as critical players for the transformation of the agricultural, chemical, energy, and pharmaceutical sectors. This Special Issue delves into the latest advancements and applications of microbial biocatalysts, shedding light on groundbreaking research that bridges fundamental science with practical innovations.

In a fascinating study, researchers explored the enzymatic potential of fucoidan-active endo-fucanase derived from the marine bacterium *Wenyingzhuangia fucanilytica*. Fucoidans, sulfated polysaccharides from brown algae, are renowned for their anticoagulant properties but present challenges due to their complex structures (contribution 1). The identified endo-fucanase, which belongs to the GH168 family [3], catalyzed specific cleavage of sulfated α-L-fucose linkages, enabling the production of high- and low-molecular-weight fucoidan derivatives with regular sulfation patterns. Notably, derivatives with 2,4-di-O-sulfation exhibited superior anticoagulant activity compared to those with only 2-O-sulfation. This research not only expands our understanding of fucanase specificity but also highlights the potential for producing standardized fucoidan preparations with enhanced therapeutic properties.

The poultry industry faces persistent challenges in optimizing feed efficiency, and microbial enzymes offer a promising solution. One study investigated the use of crude and purified fungal xylanases, derived from *Trichoderma harzianum* and *Geobacillus stearothermophilus* [4], as feed additives for chickens (contribution 2). The enzyme treatments significantly increased the yields of reducing sugars from chicken feeds, with the analysis revealing the production of beneficial xylooligosaccharides (XOSs) and monosaccharides. These hydrolysis products can modulate gut microbiota, potentially improving feed efficiency and overall poultry health. This research paves the way for the development of enzyme-based nutritional strategies that could revolutionize poultry production, offering sustainable and cost-effective alternatives to traditional feed additives.

Lipases are versatile enzymes with broad applications in industries ranging from food processing to biofuel production. One study in this Special Issue focused on a novel lipase from *Streptomyces exfoliatus*, expressed using a *Rhodococcus* host (contribution 3). The enzyme, classified within subfamily I.7 of lipolytic enzymes, exhibited optimal activity at 60 °C and pH 8.5. Its stability was enhanced in the presence of specific cosolvents and metal ions. Beyond its hydrolytic capabilities, the enzyme demonstrated the ability to degrade polyester-type polymers and catalyze the synthesis of sugar fatty acid esters. This multifunctionality highlights its potential for diverse biotechnological applications, including waste management and the synthesis of value-added products.

Heterologous protein expression remains a cornerstone of industrial biotechnology, and optimizing this process is crucial for cost-effective enzyme production [5]. One manuscript in this Special Issue tackled the challenges associated with expressing a thermophilic endoglucanase from *Thermothielavioides terrestris* in *Pichia pastoris* (contribution 4). The researchers implemented a multifactorial strategy involving genome editing, neutral loci selection, and high-density fermentation. They discovered that three copies of the expression cassette yielded the highest enzyme activity, suggesting limitations related to translational and post-translational processes. This study also demonstrated the potential of *Pichia pastoris* single-cell protein (SCP) as a sustainable protein source, highlighting the dual benefits of enzyme production and SCP generation in a single process.

The valorization of pulp and paper mill sludge (PPMS) is another critical area where microbial enzymes can drive sustainable solutions [6]. One study optimized β-glucosidase production by using *Aspergillus japonicus* for cellulose hydrolysis (contribution 5). Using a combination of experimental designs, researchers enhanced enzyme production by more than 25-fold, demonstrating its effectiveness in hydrolyzing cellulose from PPMS. Supplementing commercial cellulase cocktails with the optimized β-glucosidase further improved glucose yields, showcasing the potential for integrating microbial enzymes into industrial waste valorization processes.

Enzymatic glycosylation is a powerful tool for synthesizing bioactive compounds; one study in this Special Issue focuses on β-acuminosidase synthesized from *Penicillium multicolor*. Researchers explored its application in transglycosylating tyrosol [7], producing a mixture of glycosylated derivatives (contribution 6). This marks the first report of β-acuminosidase with the ability to glycosylate phenolic acceptors, opening new avenues for the enzymatic synthesis of complex glycosides with potential pharmaceutical applications.

Xanthan, a widely used biopolymer, presents challenges for enzymatic degradation due to its complex structure. In one study, researchers investigated the role of carbohydrate-binding modules (CBMs) in enhancing the catalytic properties of xanthanase (contribution 7). They discovered that fusing the enzyme with CBMs significantly improved its thermostability and substrate affinity, resulting in higher yields of oligoxanthan with enhanced antioxidant activity. This research lays the groundwork for the rational design of xanthan-degrading enzymes, facilitating the production of functional oligomers for industrial applications.

Rare sugars like D-allulose hold significant promise as low-calorie sweeteners with health benefits. A comprehensive review in this Special Issue discusses recent advancements in D-allulose production [8], focusing on newly identified D-allulose 3-epimerases (DAEases) with improved thermotolerance and catalytic efficiency (contribution 8). Strategies such as enzyme engineering, immobilization, and the use of microbial hosts like *Bacillus subtilis* are highlighted as key approaches for enhancing industrial-scale production. This review underscores the potential for bridging the gap between laboratory research and commercial manufacturing of rare sugars, contributing to healthier dietary options and sustainable industrial processes.

Finally, an insightful review addresses the broader context of plastic waste management in Europe [9], emphasizing the role of biotechnological tools in enhancing recycling efforts (contribution 9). While current enzymatic approaches are primarily effective on polyester-based polymers like PET, this review highlights the need for advancements in depolymerizing more recalcitrant plastics such as polyurethanes and polyolefins. Integrating chemoenzymatic technologies with optimized collection and sorting systems could significantly enhance plastic circularity, offering a sustainable alternative to conventional waste management practices.

In conclusion, the manuscripts featured in this Special Issue illustrate the transformative potential of microbial biocatalysts across diverse applications, from plastic degradation and feed optimization to therapeutic enzyme production and rare sugar synthesis. As we continue to harness the power of microbial enzymes, their integration into industrial processes promises to drive sustainability, efficiency, and innovation. The insights gained from these studies not only advance our scientific understanding but also pave the way for practical solutions to global challenges, reinforcing the pivotal role of biocatalysis in shaping a more sustainable future.

## References

[1] Katsimpouras C., Stephanopoulos G. (2021). Enzymes in Biotechnology: Critical Platform Technologies for Bioprocess Development.

[2] Tatta E.R., Imchen M., Moopantakath J., Kumavath R. (2022). Bioprospecting of Microbial Enzymes: Current Trends in Industry and Healthcare.

[3] Shen J., Chang Y., Zhang Y., Mei X., Xue C. (2020). Discovery and Characterization of an Endo-1,3-Fucanase From Marine Bacterium *Wenyingzhuangia fucanilytica*: A Novel Glycoside Hydrolase Family. Front. Microbiol..

[4] Azouz R.A.M., Hegazy U.M., Said M.M., Bassuiny R.I., Salem A.M., Fahmy A.S. (2021). Improving the catalytic efficiency of thermostble *Geobacillus stearothermophilus* xylanase XT6 by single-amino acid substitution. J. Biochem..

[5] Karnaouri A.C., Topakas E., Christakopoulos P. (2014). Cloning, expression, and characterization of a thermostable GH7 endoglucanase from *Myceliophthora thermophila* capable of high-consistency enzymatic liquefaction. Appl. Microbiol. Biotechnol..

[6] Duncan S.M., Alkasrawi M., Gurram R., Almomani F., Wiberley-Bradford A.E., Singsaas E. (2020). Paper mill sludge as a source of sugars for use in the production of bioethanol and isoprene. Energies.

[7] Potocká E., Mastihubová M., Mastihuba V. (2015). Enzymatic synthesis of tyrosol glycosides. J. Mol. Catal. B Enzym..

[8] Moroz O.V., Jensen P.F., McDonald S.P., McGregor N., Blagova E., Comamala G., Segura D.R., Anderson L., Vasu S.M., Rao V.P. (2018). Structural Dynamics and Catalytic Properties of a Multimodular Xanthanase. ACS Catal..

[9] Schmidt S., Laner D., Van Eygen E., Stanisavljevic N. (2020). Material efficiency to measure the environmental performance of waste management systems: A case study on PET bottle recycling in Austria, Germany and Serbia. Waste Manag..

